# Detection of HIV T-cell responses with polifunctionality and high plasma levels of the B-chemokine MDC in exposed HIV-seronegative individuals (ESN)

**DOI:** 10.1186/1742-4690-9-S2-P258

**Published:** 2012-09-13

**Authors:** G Turk, L Abusamra, N Laufer, AM Rodríguez, J Falivene, Y Ghiglione, A Mangano, L Giavedoni, MM Gherardi

**Affiliations:** 1 Hospital de Pediatría Juan P Garrahan, Buenos Aires, Argentina; 2 Southwest National Primate Research Center, San Antonio, TX, USA; 3 Instituto de Investigaciones Biomédicas en Retrovirus y Sida, Universi, Buenos Aires, Argentina

## Background

The mechanisms of protection from infection in ESN individuals are largely undefined and likely multifactorial. Our aim was to analyze HIV-specific T-cell responses and plasma cytokine levels in ESN and their respective HIV partners (HIV-P) in comparison with HIV chronically infected subjects (C) and healthy donors (HD).

## Methods

Plasma levels of 37 cytokines were measured by Luminex technology in: 10 HD, 9 ESN and their HIV-P, and 12 C. All HIV patients were off-HAART. Frozen PBMCs from the same individuals were used to determine HIV-specific T-cell responses by IFN-g ELISPOT. Also, production of different cytokines was evaluated in certain samples by intracellular staining (ICS). Data was compared inter- and intra-groups and correlated to viral load (VL) and CD4 T cell counts, using parametric and non-parametric statistics.

## Results

Elispot responses were evaluated in 6 of the serodiscordant couples. Two ESN showed T-cell responses against Env or Gag. ESN had a higher proportion of HIV-specific bifunctional and trifunctional cells in comparison with their HIV-P partners. HIV-P had Nef and Env T-cell responses of significant higher magnitudes compared to C. Macrophage-derived chemokine (MDC), characterized to have HIV-suppressive activities, was the only soluble marker significantly elevated in plasma of ESN in comparison with HD. Interestingly, MDC showed a positive correlation with CD4 T-cell count among HIV-P. HIV-P compared to C showed minor levels of the inflammation biomarker sCD40L associated with AIDS defining illness, and in this group VL was positively correlated with IP-10 and IL-10, whereas TNF-a correlated with VL in C.

## Conclusion

Both innate and specific immune components may be acting in the resistance to HIV infection in ESN. On the other hand, the characteristics of the immune response generated in their partners can also play a role in the viral pressure generated affecting the viral population to be transmitted.

